# Tunable Exciton Modulation and Efficient Charge Transfer in MoS_2_/Graphene van der Waals Heterostructures

**DOI:** 10.1021/acsnano.4c17354

**Published:** 2025-05-15

**Authors:** Omid Ghaebi, Tarlan Hamzayev, Till Weickhardt, Muhammad Sufyan Ramzan, Takashi Taniguchi, Kenji Watanabe, Caterina Cocchi, Domenico De Fazio, Giancarlo Soavi

**Affiliations:** † Institute of Solid State Physics, 9378 Friedrich Schiller University Jena, Jena 07743, Germany; ‡ Institute of Physics, 11233 Carl von Ossietzky Universität Oldenburg, Oldenburg 26129, Germany; § Research Center for Materials Nanoarchitectonics, 52747 National Institute for Materials Science, 1-1 Namiki, Tsukuba 305-0044, Japan; ∥ Research Center for Electronic and Optical Materials, National Institute for Materials Science, 1-1 Namiki, Tsukuba 305-0044, Japan; ⊥ Center for Nanoscale Dynamics (CeNaD), Carl von Ossietzky Universität Oldenburg, Oldenburg 26129, Germany; # Department of Molecular Sciences and Nanosystems, 19047 Ca’ Foscari University of Venice, 30172 Venice, Italy; ∇ Abbe Center of Photonics, Friedrich Schiller University Jena, Jena 07743, Germany

**Keywords:** MoS_2_, graphene, charge transfer, 2D materials, photoluminescence, exciton, trion

## Abstract

Monolayer transition metal dichalcogenides (TMDs) are direct gap semiconductors where the optical properties are dominated by strongly interacting electron–hole quasi-particles. Understanding the interactions among these quasi-particles is crucial for advancing optoelectronic applications. Here, we examine the electrical tunability of light emission from the A and B excitons in monolayer MoS_2_ and MoS_2_/graphene heterostructures and unravel the competition between the A exciton to trion formation and charge transfer processes. Our results show significant gate-tunable quenching of the photoluminescence intensity from A excitons with notable differences due to charge transfer in the heterostructure. Furthermore, we observe a distinct superlinear correlation between the A exciton photoluminescence intensity and high doping levels in MoS_2_, which continues until the density of photoexcited excitons exceeds and saturates the free carrier density. This phenomenon ceases to occur in MoS_2_/graphene, where MoS_2_ remains almost undoped across all values of the applied external voltage. In contrast, the B exciton photoluminescence is unaffected by doping in MoS_2_, while it decreases analogously to that of the A excitons in the MoS_2_/graphene heterostructure, indicating the relevance of gate-tunable charge transfer from hot electrons before any internal recombination.

## Graphical abstract

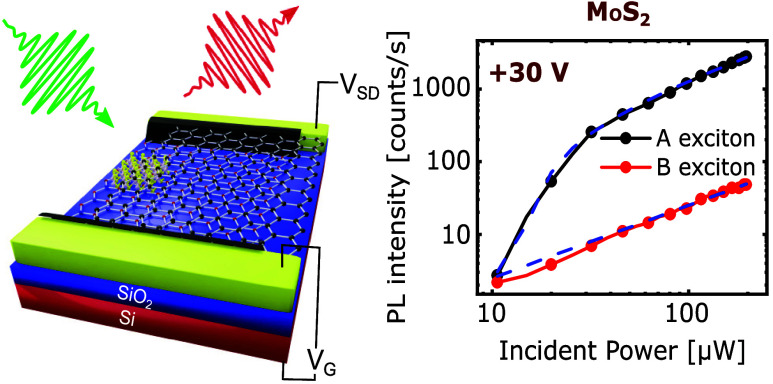

Monolayer transition metal dichalcogenides (TMDs) are direct gap semiconductors in the ±K valleys,^1–4^ and their optical properties are dominated by strongly bound electron–hole pairs (excitons).^5^ Furthermore, in the presence of doping, excitons can couple to a free electron (hole) to form negatively (positively) charged excitons, also known as trions. Since trions are characterized by ultrafast nonradiative recombination dynamics,^6^ the amount of doping in a TMD monolayer strongly affects the excited state lifetime and, as a consequence, the photoluminescence (PL) quantum yield^6^ and the efficiency of nonlinear processes such as exciton–exciton annihilation^7–9^ and harmonic generation.^10,11^ This directly impacts the performance of TMD-based optoelectronic devices, such as photodetectors and light-emitting diodes.^12,13^ Therefore, the deterministic control of doping in TMDs has both fundamental and technological relevance. So far, the main approaches for tuning doping in TMDs have been chemical,^7,13–15^ electrostatic doping,^16–18^ and active filtering by charge transfer to graphene.^19^ Recently, ultrafast all-optical trion dissociation with THz pulses has also been demonstrated.^20^

In this work, we study and compare the effect of electrostatic doping and excitation fluence on the optical properties of pristine monolayer MoS_2_ and a MoS_2_/graphene heterostructure (HS). We focus on the gate and power dependence of the PL in both samples for both the A and B excitons, and we identify and discuss the fingerprints of trion saturation (in pristine MoS_2_) and charge transfer (in the HS) based on the following experimental observations: (1) In doped MoS_2_ (i.e., when the total free carrier density is *n*_D_ ≫ 10^11^ cm^–2^), the PL intensity scales superlinearly with the incident power^21^ until all the free electrons are saturated by the photoexcited excitons.^20^ This effect is absent in the HS, where the (static) charge transfer keeps the doping required for the superlinear power dependence relatively low (*n*_D_ ≤ 10^11^ cm^–2^) at any value of the gate voltage; (2) B excitons are much less affected by doping compared to A excitons in MoS_2_ due to the ultrafast internal recombination, which dominates over all other relaxation pathways. In contrast, in the HS, the B excitons undergo a gate-tunable modulation similar to that of the A excitons, indicating that a fraction of the charge transfer in the HS occurs from hot electrons (i.e., before internal recombination).

With these findings, our work provides new insights into the photophysics of gate-tunable TMDs and related HSs and thus offers a guide to design nanoscale optoelectronic devices such as gate-tunable light-emitting diodes.^22^

## Results and Discussion

All of the optoelectronic measurements presented in this work were performed on a single back-gated device based on monolayer MoS_2_/graphene, encapsulated in two ∼10 nm-thick hBN layers. This device allows one to perform optical measurements on both the monolayer MoS_2_ region and the MoS_2_/graphene HS (Figure 1). For the fabrication, we used the method described in refs 23,24 . Monolayer graphene, monolayer MoS_2_, thin hBN, and graphite flakes (used for the electrical contacts) were exfoliated from synthetic bulk crystals using Scotch tape. Subsequently, the layers were picked up and stacked using a thin stamp of polycarbonate (PC) and eventually transferred to a silicon wafer coated with a ∼90 nm layer of thermally grown silicon dioxide (SiO_2_) with pre-patterned Au/Cr contacts (see Methods Section for details). The device effectively behaves as a graphene field-effect transistor (FET), where a monolayer of MoS_2_ partially covers the graphene channel (see Supporting Information S1 for characterization details). Notably, only the graphene monolayer is directly in contact with the source and drain electrodes, while the MoS_2_ flake is in contact only with graphene (see panel c in Figure 1). For simplicity, throughout this article, we refer to the regions of hBN/MoS_2_/hBN and hBN/MoS_2_/graphene/hBN as MoS_2_ and HS, respectively.

**Figure 1:**
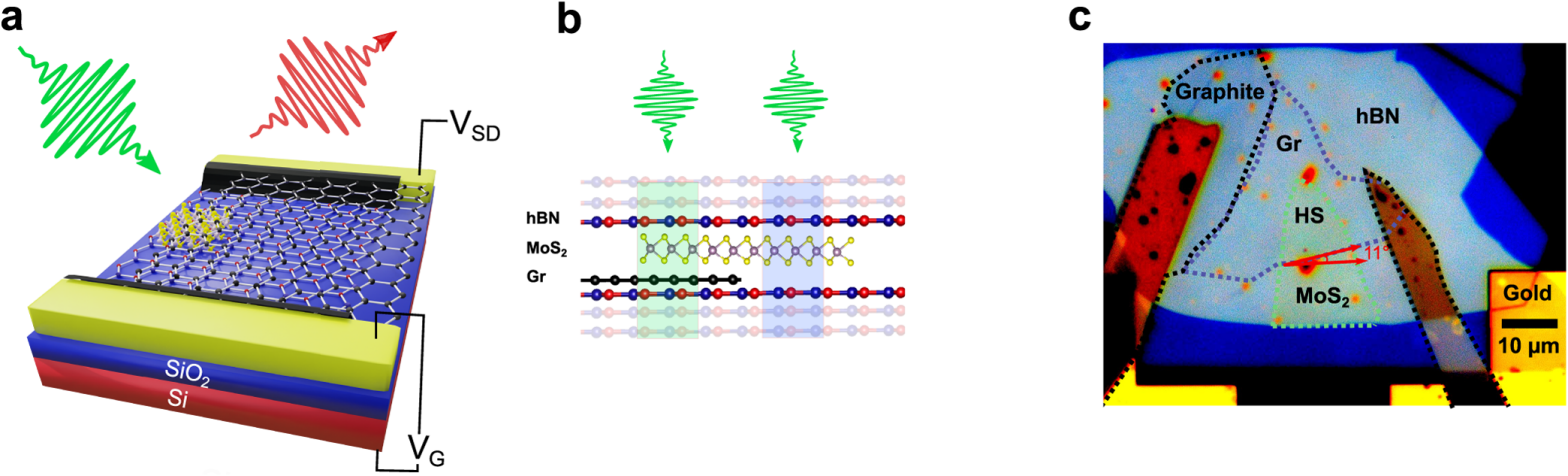
MoS_2_ and MoS_2_/graphene HS device. (a) Sketch of the gated HS. The device consists of a double hBN-encapsulated monolayer MoS_2_/monolayer graphene connected to gold contacts via graphite flakes (black plates). *V*_G_ and *V*_SD_ represent the gate and source-drain voltages, respectively. (b) Side view of the HS. Two regions of interest include MoS_2_ (light-blue rectangle) and the MoS_2_/graphene HS (green rectangle). (c) Microscope optical image of the device consisting of graphene (light-blue dashed area), MoS_2_ (green dashed area), and the related HS. The dashed black area corresponds to graphite flakes used as Ohmic contacts. The red arrows show the angle between MoS_2_ and graphene edges, corresponding to the crystallographic zigzag directions. The device operates as a graphene field-effect transistor where only the graphene layer is in direct contact with the source and drain electrodes.

Figure 2 shows exemplary PL spectra recorded on MoS_2_ (panels a and c) and the HS (panels b and d) for two gate voltages (*V*_G_) of −30 and 30 V and fixed input powers of ∼10 and ∼160 μW (see Methods Section for experimental details). Here, we can immediately appreciate some key results that will be the focus of the following discussion: (i) At – 30 V, the PL intensity of the A exciton in the HS is about 1 order of magnitude lower compared to MoS_2_ (see black curves in Figure 2 panels a vs b, and panels c vs d); (ii) the PL from the B exciton is clearly visible in the HS (panels b and d), while it is significantly smaller in MoS_2_ (panels a and c); (iii) in MoS_2_, the trion peak is only visible at ∼10 μW of incident power (panel c) when *V*_G_ increases from – 30 to 30 V, while exciton to trion conversion is negligible when tuning *V*_G_ = −30 to 30 V at ∼160 μW; (iv) a comparison of the normalized spectra of MoS_2_ and HS at *V*_G_= 30 V and ∼10 μW (panel f) clearly shows the conversion of trions to A excitons (blue-shift) with the introduction of graphene (i.e., in the HS), while the conversion of trions to A excitons is much weaker at ∼160 μW (panel e).

**Figure 2:**
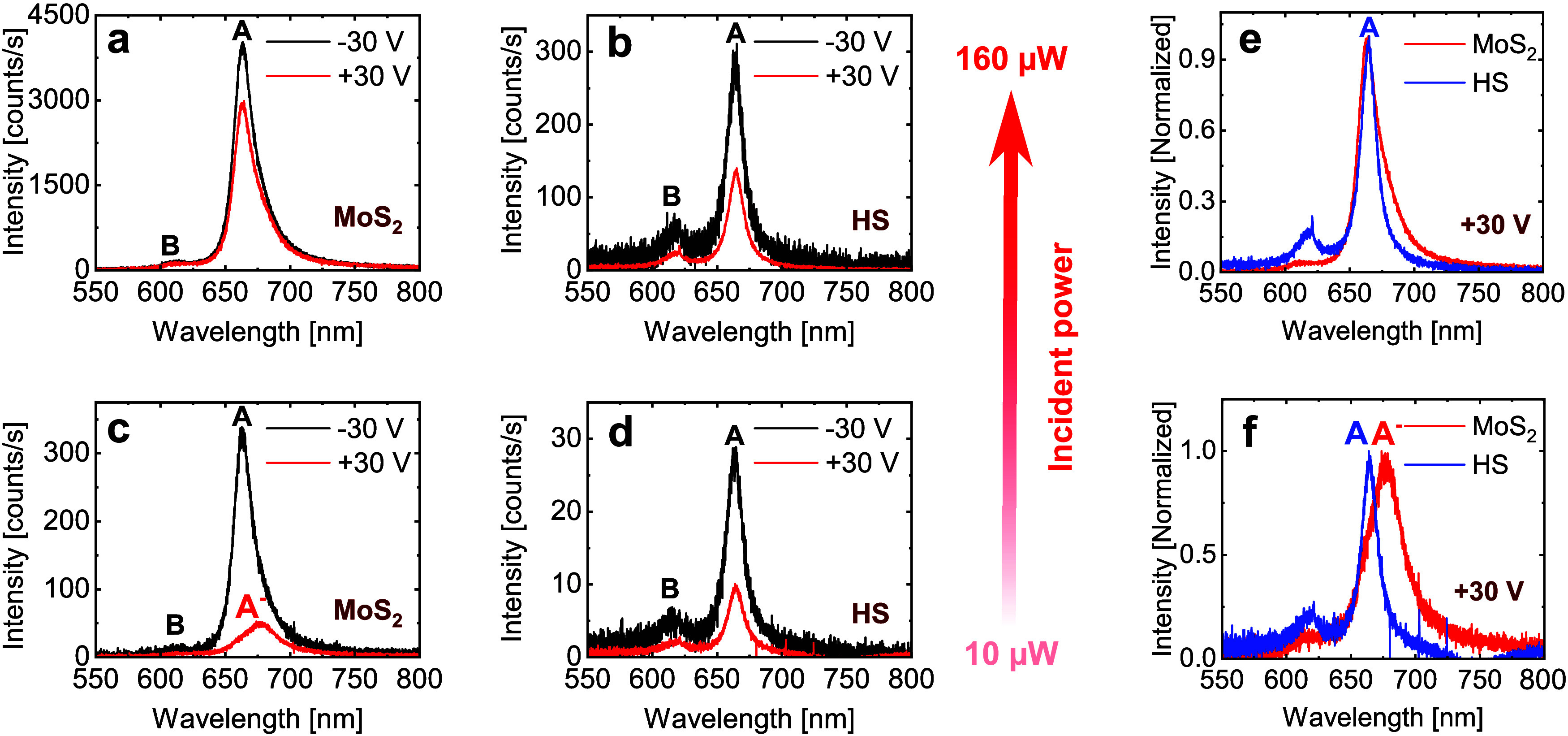
Gate-dependent PL in MoS_2_ and HS. (a, c) Gate-dependent PL spectra in MoS_2_ at an incident power of ∼160 and ∼10 μW, respectively. (b, d) Gate-dependent PL spectra in the HS at an incident power of ∼160 and ∼10 μW, respectively. (e, f) Normalized PL spectra in MoS_2_ (red) and HS (blue) at incident power of ∼160 and ∼10 μW, respectively, and *V*_G_ = 30 V.

From a qualitative viewpoint, some of these differences can be rationalized by considering the physical processes responsible for the PL tuning in MoS_2_ and in the HS. For example, at −30 V, MoS_2_ is weakly doped (*n*_D_ ≤ 10^11^ cm^–2^), and its PL is dominated by a bright emission from the A exciton. Increasing *V*_G_ (and thus the electron doping) reduces the overall PL intensity and red-shifts the emission wavelength^16^ (Figure 2c). In contrast, in the HS, the presence of graphene forces two additional effects: (1) steady-state charge transfer from MoS_2_ to graphene removes the excess doping and makes MoS_2_ relatively undoped at any value of the external *V*_G_;^19,25^ (2) ultrafast charge transfer from the bottom of the conduction band reduces the PL intensity of the A exciton by ∼1 order of magnitude.^26^ However, this explanation does not account for some of our experimental observations. For instance, in MoS_2_, efficient trion formation occurs only at low values of the incident power (∼10 μW). Additionally, there is a different response of A excitons and B excitons to trion formation and incident power in both MoS_2_ and the HS. Thus, in the following, we take advantage of both electrical gating and charge transfer to graphene to further explore the photophysics and interplay between neutral excitons and trions in MoS_2_ and in the HS.

To gain a deeper understanding of the interplay between neutral A and B excitons and trions, we performed excitation power dependence measurements on both monolayer MoS_2_ and the HS (Figure 3). Specifically, we tune the incident power in the range ∼10–200 μW for *V*_G_ = −30, 0, and 30 V, and we record the PL spectra from 600 to 750 nm to detect both A and B excitons, isolated by Gaussian fitting.

**Figure 3:**
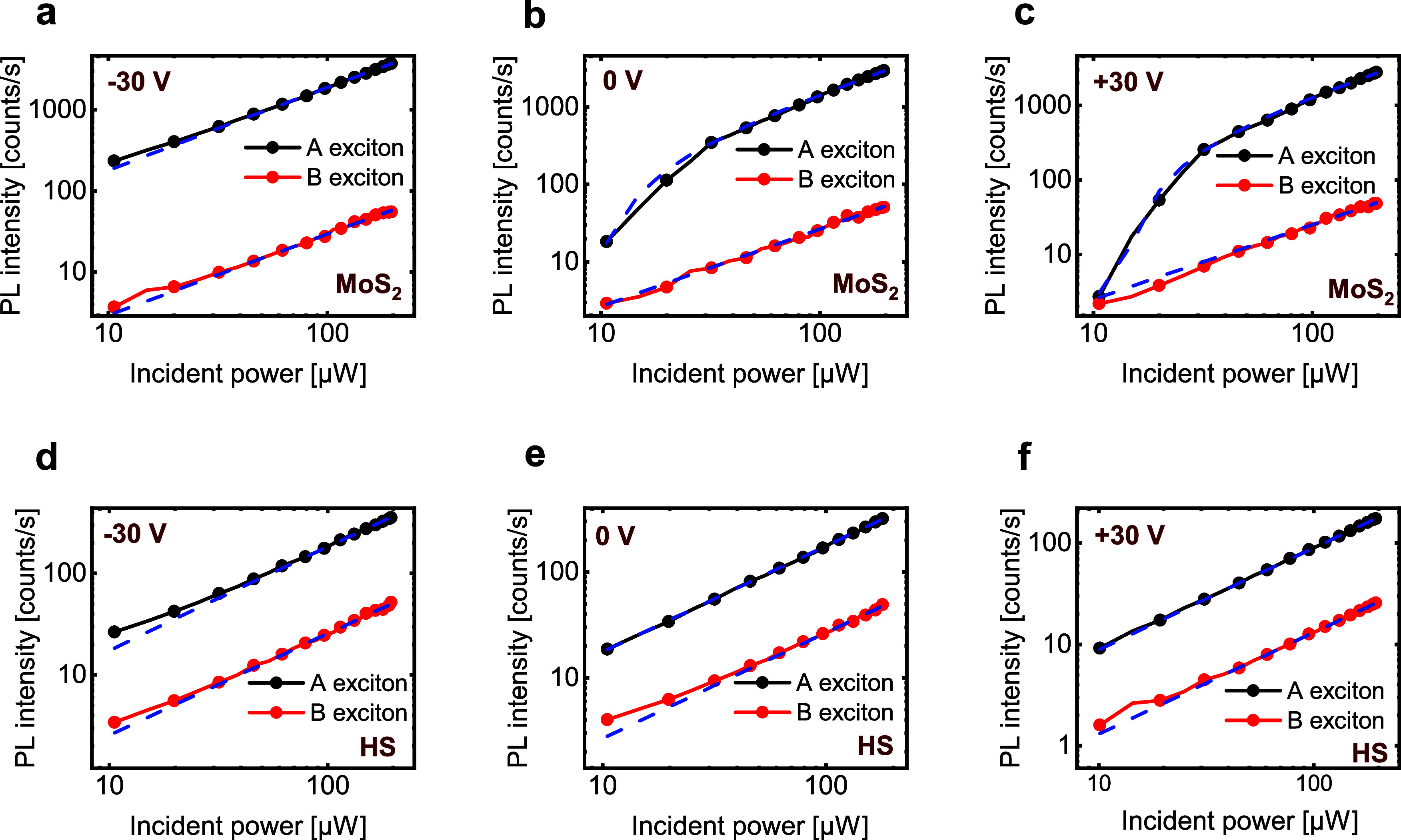
Power-dependent PL in MoS_2_ and in the HS. (a–c) Power-dependent PL for *V*_G_ = −30, 0, and 30 V in monolayer MoS_2_. (d–f) Power-dependent PL for *V*_G_ = −30, 0, and 30 V in the HS.

The PL intensity in monolayer MoS_2_ can be controlled via *V*_G_, which can be used to tune the doping and thus the gate-induced free carrier density *n*_G_.^6,16,17^ Note that *n*_D_ is the sum of *n*_G_ and the unintentional doping density in MoS_2_. In addition, the incident laser power can be used to control the neutral exciton to trion conversion process.^21^ When MoS_2_ is weakly doped (*n*_D_ ≤ 10^11^ cm^–2^), the PL intensity scales linearly with the incident power (Figure 3a). Instead, by increasing *V*_G_ (and accordingly *n*_D_), we observe a superlinear dependence of the A exciton PL intensity as a function of the incident power (panels b and c in Figure 3). This superlinear power dependence of the PL intensity is present until the photoexcited A exciton density (*n*_A_) is equal to or higher compared to *n*_D_, and after this threshold value, the PL intensity goes back to a linear dependence with respect to the input power.^20,21^ Consequently, the interplay between *n*_D_ and *n*_A_ directly controls the superlinear behavior of the A exciton PL intensity.^21^

Interestingly, the PL intensity from the HS (panels d–f in Figure 3) scales linearly with the input power for any value of *V*_G_. This clearly indicates the dual effect enabled by the presence of graphene in the HS: efficient trion filtering^19^ and screening of the electric field from the back gate.^27^ As a consequence, MoS_2_ remains undoped for any value of the external *V*_G_. This is in agreement with the observation in Figure 2 that the PL spectra of the HS do not show any trion footprint while tuning *V*_G_.^19^

To further clarify the process of neutral A exciton → trion conversion, we implement a model based on rate equations for the A, B, and trion densities to fit the power-dependent PL results^21^

1 $$\frac{dn_{A}}{dt}=\frac{\eta_{A}p}{E_{\mathrm{in}}S}-\frac{n_{A}}{\tau_{A}}-kn_{A}\left(n_{D}-n_{A^{-}}\right)+\frac{n_{A^{-}}}{\tau_{\mathrm{diss}}}+\frac{n_{B}}{\tau_{B\to A}}$$

2 $$\frac{dn_{A^{-}}}{dt}=kn_{A}\left(n_{D}-n_{A^{-}}\right)-\frac{n_{A^{-}}}{\tau_{A^{-}}}-\frac{n_{A^{-}}}{\tau_{\mathrm{diss}}}$$

3 $$\frac{dn_{B}}{dt}=\frac{\eta_{B}p}{E_{\mathrm{in}}S}-\frac{n_{B}}{\tau_{B}}-\frac{n_{B}}{\tau_{B\to A}}$$

Equations 1, 2, and 3 describe the temporal evolution of the exciton densities for the neutral A and B excitons (*n*_A_, *n*_B_) and for trions (*n*_A^–^_). The variables η, *p*, *E*_in_, S, *n*_D_, *k*, τ_A_, τ_A^–^_, τ_B_, τ_diss_, and τ_B→A_ describe the absorption efficiency, incident power, input photon energy, laser spot size area, free carrier density, trion formation coefficient, A exciton lifetime, trion lifetime, B exciton lifetime, trion dissociation time, and B exciton to A exciton internal recombination time, respectively. In eqs 1 and 3, we considered the absorption efficiency η_A_, η_B_ as independent of *V*_G_ for pristine MoS_2_. While this assumption would not hold true for absorption close to the A and B exciton resonances,^16,28^ the absorption at 532 nm (2.33 eV) is independent of *V*_G_, as reported for instance in ref 28. In contrast, tuning *V*_G_ from −30 to 30 V in the HS results in a similar reduction by approximately a factor of 2 for both η_A_ and η_B_, as we discuss in detail in the following. We explain this result as a gate-tunable hot-electron transfer from MoS_2_ to graphene (see panel d in Figure 4), as discussed for instance in ref 29.

**Figure 4:**
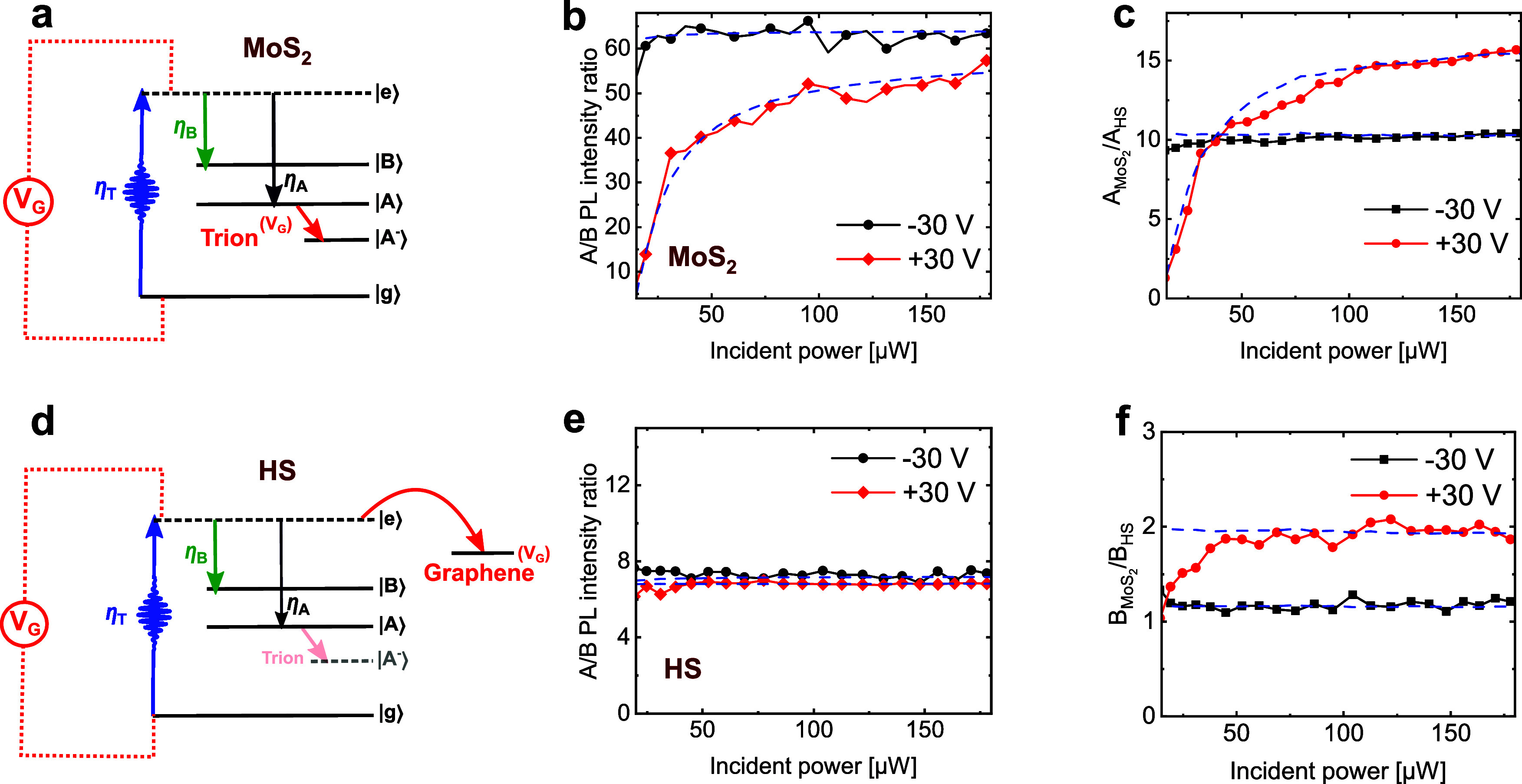
PL intensity ratios in MoS_2_ and HS. (a, d) Impact of *V*_G_ on trion formation and charge transfer in monolayer MoS_2_ and HS. (b, e) Power-dependent A/B exciton PL intensity ratio for *V*_G_ = −30 and 30 V in monolayer MoS_2_ and HS. (c, f) Power-dependent $\frac{A_{{\mathrm{MoS}}_{2}}}{A_{\mathrm{HS}}}$ and $\frac{B_{{\mathrm{MoS}}_{2}}}{B_{\mathrm{HS}}}$ PL intensity ratio for *V*_G_ = −30 and 30 V.

The first term on the left-hand side in eqs 1 and 3 defines the exciton photogeneration rate, while the other terms describe the different types of radiative and nonradiative recombination, which are mainly responsible for the total PL emission and quantum yield of the A and B excitons. The superlinear power dependence reported in Figure 3b,c can be explained from the third term in eq 1, which defines the conversion process from the A excitons to trions, depending on *n*_D_. Furthermore, the last terms in eqs 1 and 3 represent the fast internal recombination channel of B to A excitons. It is worth noting that effects such as intervalley scattering^30–32^ are not included in our model for two reasons: (i) intervalley scattering only changes the relative exciton density between the valleys, without affecting the total excited state density; (ii) in our experiments, we use linearly polarized light, which ideally gives a homogeneous distribution of the valley excitons. By finding the steady-state solutions of differential eqs 1, 2, and 3 and optimizing solutions by *k*, τ_A_, τ_A^–^_, τ_diss_, τ_B_, τ_B→A_, η_A_, η_B_, and *n*_D_ values to fit the experimental data in Figure 3 (blue dashed lines), we obtain for MoS_2_ *n*_D_ ≤ 9 × 10^10^ cm^–2^, *n*_D_ ∼ 9.8 × 10^11^ cm^–2^, and *n*_D_ ∼ 2.3 × 10^12^ cm^–2^ for *V*_G_ = −30, 0, and 30 V, respectively (see Supporting Information S2 for details). We note that our fitting function returns *n*_D_ ∼ 9 × 10^10^ cm^–2^ as the upper limit of doping. Namely, in our experiments, we are able to detect a linear PL power dependence for any *n*_D_ smaller than this threshold. In contrast, by considering the absence of trions PL in the HS (panels b and d in Figure 2) and looking at the first term in eq 2, we can see that once *n*_A^–^_ = 0, the interaction between trions and *n*_D_ disappears, and changing *n*_D_ will not lead to any superlinear behavior in the HS (panels d–f in Figure 3). This confirms that static charge transfer from MoS_2_ to graphene makes MoS_2_ undoped. The value of *n*_D_ ∼ 2.3 × 10^12^ cm^–2^ at *V*_G_ = 30 V in MoS_2_ obtained from this fit is in good agreement with *n*_D_ ∼ 1 × 10^12^ cm^–2^ to *n*_D_ ∼ 1 × 10^13^ cm^–2^ reported in refs 6,16,33 for similar devices. Finally, we note that the results presented in Figure 3 involve several fitting parameters. Since the values obtained from the fitting are not uniquely defined, one should consider them more for a qualitative description of the physical phenomena at play rather than for a precise quantification of the observed effects. Still, we note that in MoS_2_ at *V*_G_ = 30 V, we obtain from the fitting a lifetime for the A exciton of τ_A_ = 30 ns, in good agreement with results reported in the literature.^6,34–38^

The PL emission in MoS_2_ and HS is governed by two dominant mechanisms: trion formation due to conversion from neutral excitons (in MoS_2_) and charge transfer to graphene (in the HS). While both effects have been widely studied in relation to the A exciton PL intensity,^19,21,33,39^ their impact on the B exciton has been less investigated. In our experiments, the PL intensity from the B exciton displays a linear power dependence for all of the investigated values of input power and *V*_G_ for both MoS_2_ and the HS (Figure 3). This clearly points toward the fact that B excitons are almost unaffected by electrostatic doping.

Next, we examine the A to B exciton PL intensity ratios in MoS_2_ and in the HS at *V*_G_ = −30 and 30 V at different values of the input power (panels b and e in Figure 4). The A/B PL intensity ratio has often been used as an indirect probe of the defect density in monolayer TMDs^40^ due to the shorter lifetime of B excitons, which minimizes the impact of nonradiative decay pathways, leaving their PL intensity almost independent of defect density. In contrast, the PL intensity of the A excitons is strongly affected by the presence of defects.^40^ Here, we follow the same approach: B excitons undergo faster decay dynamics, and they are thus less affected by other relaxation mechanisms due to fast internal recombination to A excitons, for instance, due to defects,^36^ charge trapping,^41^ and exciton–exciton annihilation.^8^ In our experiment, the additional nonradiative decay channel is provided by ultrafast charge transfer to graphene. We thus look at the ratio of PL intensities to investigate the impact of charge transfer and trion formation on the optoelectronic response of our device.

In MoS_2_ at *V*_G_ = −30 V, the A/B PL intensity ratio is ∼60 for any value of the incident power (Figure 4b). This can be understood considering that in MoS_2_, when *n*_D_ ≤ 10^11^ cm^–2^, both the A and B excitons have a linear dependence versus the input power (Figure 3a); thus, their ratio remains constant. In contrast, at *V*_G_ = 30 V (*n*_D_ ∼ 2.3 × 10^12^ cm^–2^), the A/B PL intensity ratio increases and saturates between 14 and 57 for incident power between ∼20 and ∼180 μW (Figure 4b). Again, this is a consequence of the crossover from superlinear to linear power dependence of the A exciton PL intensity reported in Figure 3c. Here, we highlight that MoS_2_ shows a low value (14) of the A/B PL intensity ratio only upon doping (*V*_G_ = 30 V) and for low values of the input power, namely when the trion density is larger compared to the neutral A exciton density. In this regard, trions play the same role as defects in ref 40, namely they quench the neutral A exciton PL emission while leaving the B exciton unaffected.

In contrast, the A/B intensity ratios in the HS are ∼6–7 and independent of power for both *V*_G_ = −30 V and *V*_G_ = 30 V (Figure 4e). From this, we can infer that the A/B PL intensity ratio is always lower compared to that of the pristine MoS_2_ sample, regardless of *V*_G_ and input power. This difference can be ascribed to the efficient charge transfer from the bottom of the conduction band of MoS_2_ to graphene,^19,26^ a process that affects only the A exciton PL intensity and thus reduces the overall A/B ratio. However, as we discuss in the following, there is another mechanism at play. To study this, we focus on the ratios of the PL intensities for the A and B excitons in MoS_2_ versus the HS (panels c,f in Figure 4).

Panel c in Figure 4 shows the A exciton PL intensity ratios of MoS_2_ to the HS at *V*_G_ = −30 V (black squares) and 30 V (red circles). The ratio is ∼10 and independent of power at *V*_G_ = −30 V, namely when *n*_D_ ≤ 9 × 10^10^ cm^–2^. When *n*_D_ ∼ 2.3 × 10^12^ cm^–2^ (*V*_G_ = 30 V), the ratio goes from ∼1 to ∼14 depending on the input power. This result implies fundamental aspects connected to the internal recombination pathways in MoS_2_ and the HS: (1) At *n*_D_ ≤ 10^11^ cm^–2^, the charge transfer from MoS_2_ to graphene accounts for a factor of ∼10 reduction in the PL intensity, in agreement with previously reported results.^26^ (2) In highly doped MoS_2_ (*n*_D_ ≫ 10^11^ cm^–2^), the quenching of the PL induced by the presence of trions is close to that induced by charge transfer to graphene in the HS. Namely, the PL emission from a doped MoS_2_ (at low power, where trions dominate) is equal to that of the HS. This underscores that nonradiative trion recombination and charge transfer to graphene must occur on similar time scales as reported, for instance, by refs 19,26 .

The PL intensity ratios of MoS_2_ to the HS for the B exciton reveal a completely different situation: the ratio remains constant, close to ∼1 and ∼2 (Figure 4f), at *V*_G_ = −30 V and *V*_G_ = 30 V, respectively. Considering that in TMDs the A and B excitons arise from the spin–orbit splitting of the valence band, the above observation can be attributed to one of the two possible scenarios: (i) When graphene is n-doped (*V*_G_ = 30 V), the holes can transfer from B excitons in MoS_2_ to graphene. This efficient hole transfer should introduce different quenching factors for the A and B excitons. This would correspond, for instance, to a change in τ_*B*_ in eq 3. (ii) After photoexcitation and before internal recombination, the photoexcited hot electrons are transferred to graphene. In this case, we would expect a similar modulation of the intensity for both A and B excitons. This would correspond, for instance, to a similar change to η_A_ and η_B_ as a function of *V*_G_ in eqs 1 and 3. In other words, this would account for a gate-tunable charge transfer from MoS_2_ to graphene that is almost identical for the A and B excitons.

By comparing panels b and d in Figure 2, it is evident that both A and B excitons in the HS are quenched by a factor of ∼2 by increasing *V*_G_ from – 30 to 30 V. The latter can also be seen in panel e of Figure 4, where the A/B intensity ratio remains constant over two extremes of *V*_G_. This highlights that the ultrafast charge transfer in the HS will impact primarily the A excitons, with a one order of magnitude reduction in their PL intensity (Figure 2), with an additional gate-tunable hot-electron transfer, which, at *V*_G_ = 30 V, affects both A and B excitons in the same way.

Finally, we highlight that other reports in the literature^42^ have discussed the importance of the twist angle for charge transfer in MoS_2_/graphene heterostructures. To account for this additional degree of freedom, we estimated the twist angle in our HS by comparing the edges (corresponding to the crystallographic zigzag direction) of MoS_2_ and graphene.^43^ From this, we find an angle of ≈11° (see Figure 1c), at which we do not expect any exotic behavior arising from moiré physics and/or alignment of the K bands. To support this assumption, we performed a more detailed analysis of the effect of local stacking domains on the electronic structure of the HS using density functional theory (DFT) (see details in Supporting Information S3). Specifically, we calculated the band structure and density of states of the HS considering different registries corresponding to varying twist angles between MoS_2_ and graphene, and for all configurations, we did not find any substantial differences (see Figure S2 in Supporting Information). Since the excitonic properties and the optical response of crystals are primarily dictated by their electronic structure, we can assume that registry variations between MoS_2_ and graphene do not significantly modify the current interpretation of the experimental findings.

## Conclusions

We studied gate-dependent and power-dependent PL emission from A excitons, B excitons, and trions in a field-effect device consisting of monolayer MoS_2_ and MoS_2_/graphene regions. First, by tuning both electrical doping and charge transfer to graphene, we observed a reduction of the A exciton PL intensity in the HS compared to MoS_2_ by about 1 order of magnitude. This is due to ultrafast charge transfer from the bottom of the conduction band in MoS_2_ to graphene.^26^ Second, we reported a specific superlinear power dependence of the A exciton PL intensity at large values of the doping in MoS_2_, which persists until the density of photoexcited excitons overcomes the free electron density (doping). This effect disappears in the HS, where MoS_2_ remains almost undoped at any value of *V*_G_ due to the dual functionality of graphene, namely its effective trion filtering capabilities^19^ and its ability to screen the electric field from the back gate.^27^ Finally, our analysis of the A/B exciton PL intensity ratios in MoS_2_ and HS reveals key information about how the exciton dynamics are influenced by charge transfer. In doped MoS_2_, the A/B exciton ratio changes with both doping and incident power because the A exciton is strongly affected by *V*_G_ due to trion formation, while the B exciton is insensitive to doping.^40^ In contrast, in the HS, the A/B ratio is almost independent of both *V*_G_ and power, indicating the presence of hot-electron transfer. Finally, our first-principles results do not indicate any substantial modification of the electronic structure in the HS induced by different local stacking domains. Together, these findings underscore the complex interplay of doping, exciton modulation, and charge transfer in gated layered materials and related heterostructures. These findings are thus highly relevant to designing the next generation of TMD-based optoelectronic devices.

## Methods

### Device Fabrication

The monolayer graphene flake was mechanically exfoliated from bulk synthetic graphite (HQ-graphene) by using Scotch tape (Minitron). The hBN layers and graphite contacts were exfoliated on poly­(dimethylsiloxane) (PDMS) and subsequently transferred to the Si/SiO_2_ substrate. The thicknesses of the hBN flakes were estimated by optical color contrast following the approach described in ref 44. A thin stamp made of polycarbonate (PC) on a glass slide was fabricated^23,24^ and then used to pick up the hBN layers, graphite contacts, and graphene with the help of a commercial transfer stage (HQ-graphene). Afterward, the layers were transferred onto a silicon wafer (90 nm SiO_2_) with pre-patterned gold contacts. After the PC was cleansed using a chloroform solution, the electrical wires were connected.

### Experimental Setup

The gate-dependent PL measurements were performed at room temperature using a home-built microscope with a 532 nm excitation wavelength continuous-wave laser (Cobolt 08-DPL). The PL spectra were recorded by using a spectrometer consisting of a monochromator (Horiba iHR 550) and an electrically cooled Si detector (Horiba Synapse EMCCD Camera). Electrical gating was performed by connecting the device to a source meter unit (2614B, Keithley).

## Supplementary Material



## Notes

Electrical characterization; twist angle characterization; and computational methods (PDF)
