# Supplementary material for: Tunable Exciton Modulation and Efficient Charge Transfer in MoS2/Graphene van der Waals Heterostructures
Source: ACS Nano. 2025 May 15;19(20):19027–34. doi: 10.1021/acsnano.4c17354 (PMC12120984; doi:10.1021/acsnano.4c17354)
Supplement: Supplementary file 1 [file nn4c17354_si_001.pdf]

**SUPPORTING INFORMATION**

**TITLE**

Tunable Exciton Modulation and Efficient Charge Transfer in $MoS_2$/graphene van der Waals Heterostructures

**AUTHOR LIST**

Omid Ghaebi[1,*], Tarlan Hamzayev[1,*], Till Weickhardt[1], Muhammad Sufyan Ramzan[2], Takashi Taniguchi[3], Kenji Watanabe[4], Caterina Cocchi[2,5], Domenico De Fazio[6], Giancarlo Soavi[1,7†]

**AFFILIATIONS**

[1]Institute of Solid State Physics, Friedrich Schiller University Jena, Jena, 07743, Germany

[2]Institute of Physics, Carl von Ossietzky Universität Oldenburg, 26129, Oldenburg, Germany

[3]Research Center for Materials Nanoarchitectonics, National Institute for Materials Science, 1-1 Namiki, Tsukuba 305-0044, Japan

[4]Research Center for Electronic and Optical Materials, National Institute for Materials Science, 1-1 Namiki, Tsukuba 305-0044, Japan

[5]Center for Nanoscale Dynamics (CeNaD), Carl von Ossietzky Universität Oldenburg, 26129, Oldenburg, Germany

[6]Department of Molecular Sciences and Nanosystems, Ca' Foscari University of Venice, 30172, Venice, Italy

[7]Abbe Center of Photonics, Friedrich Schiller University Jena, Jena, 07743, Germany

[*]These authors contributed equally to this work.

[†]Corresponding author: giancarlo.soavi@uni-jena.de

## S1 ELECTRICAL CHARACTERIZATION

After fabrication, we characterized the graphene field effect transistor (FET) using transconductance measurements, namely, $I_{\mathrm{SD}} - V_{\mathrm{G}}$ measurement (Fig. S1) where $I_{\mathrm{SD}}$ and $V_{\mathrm{G}}$ are source-drain current and gate voltage, respectively. From this, we estimated the carrier mobility, contact resistance, and residual charge density. The device consists of a $\sim 30\,\mu\mathrm{m} \times \sim 34\,\mu\mathrm{m}$ monolayer graphene and $MoS_2$ flakes which are encapsulated between two $\sim 10\,\mu\mathrm{m}$ thick hBN flakes. To estimate the carrier mobility for both electrons and holes, contact resistance, and residual charge density, we fit the transconductance data with the following equation[1]:

$$R = R_0 + \frac{L}{W} \cdot \frac{1}{e \cdot \mu \cdot \sqrt{n_0^2 + \left(\frac{c_{\mathrm{d}} \cdot (V_{\mathrm{G}} - V_{\mathrm{CNP}})}{e}\right)^2}} \tag{1}$$

where $L$ and $W$ define the size of the channel (graphene flake) and $R$, $R_0$, $\mu$, $n_0$, $c_{\mathrm{d}}$ and $V_{\mathrm{CNP}}$ represent the total resistance ($R = V_{\mathrm{D}}/I_{\mathrm{D}}$), contact resistance, carrier mobility, residual charge density, geometrical capacitance, and the $V_G$ at the charge neutrality point (CNP), respectively. Note that we assume a capacitance governed by the geometrical capacitance only, *i.e.*, all $V_{\mathrm{G}}$ drops on graphene and the effect of the top $MoS_2$ flake is negligible. As a result, $c_{\mathrm{d}}$ is the series capacitance per unit area of two dielectrics, namely, hBN and $SiO_2$. Considering the dielectric constants of hBN ($\varepsilon_{hBN}$=3.76) and $SiO_2$ ($\varepsilon_{\mathrm{SiO_2}}$=3.9), we estimate $c_{\mathrm{d}}$ as $3 \times 10^4\,\mathrm{F/m^2}$. By fitting the transconductance data, we found the following results for holes:

$$\begin{aligned} n_0 &= 2.5 \times 10^{11}\,\mathrm{cm^{-2}} \\ \mu &= 1 \times 10^{4}\,\mathrm{cm^2/(V\,s)} \\ R_0 &= 5.2 \times 10^{2}\,\Omega \end{aligned} \tag{2}$$

and for electrons:

$$\begin{aligned} n_0 &= 3 \times 10^{11}\,\mathrm{cm^{-2}} \\ \mu &= 9.3 \times 10^{3}\,\mathrm{cm^2/(V\,s)} \\ R_0 &= 6.2 \times 10^{2}\,\Omega \end{aligned} \tag{3}$$

We note that $\mu$ is dominated by the graphene channel between the source and drain electrodes. The measured mobility of about $1 \times 10^4\,\mathrm{cm^2/(V\,s)}$ is in excellent agreement with the typical values of mobility for hBN-encapsulated graphene FETs reported at room temperature (see *e.g.*, Refs.[2,3]).

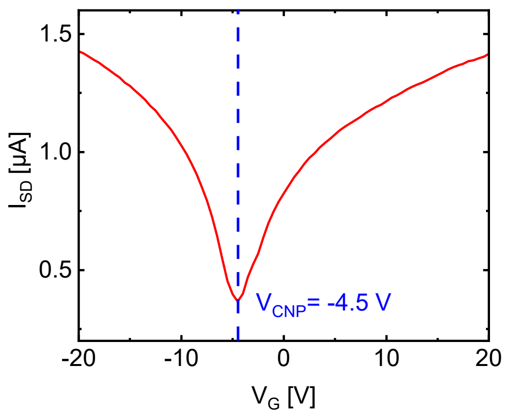


FIG. S1. $I_{\mathbf{SD}} - V_{\mathbf{G}}$ **of the device.** The blue dashed line represent $V_{\text{CNP}}$=−4.5 V.

## S2 FIT OF THE POWER-DEPENDENT PHOTOLUMINESCENCE

To fit the experimental values of the photoluminescence (PL) intensities for different values of the incident power, we use Eq.1, 2 and 3 defined in the main text. Since we did not perform any time-resolved measurements, accessing the exact values for excitonic lifetimes is not possible. Therefore, we take lifetimes as variables in the fitting procedure. Furthermore, direct estimation of $n_{\text{D}}$ is not possible in the device used in this study since $MoS_2$ is not isolated in the FET configuration *i.e.*, $MoS_2$ is not connected to both source and drain contacts. Therefore, it is best to start by fitting the PL power-dependent results in $MoS_2$ at $V_{\text{G}}$ =30 V and by taking some parameters as constants, we extract the following values in Table I:

As the next step, by fixing all the other parameters, At $V_{\text{G}}$ =0 V, we found $n_{\text{D}} \sim 9.8 \times 10^{11}\,\text{cm}^{-2}$ and then the lower bound value of $n_{\text{D}}$ in $MoS_2$ for $V_{\text{G}}$ =−30 V as $n_{\text{D}} \leq 9 \times 10^{10}\,\text{cm}^{-2}$. It is worth noting that any lower value for $n_{\text{D}}$ at $V_{\text{G}}$ =−30 V will lead to a similar power-dependent trend.

In the HS, we have the additional effect of ultrafast charge transfer from $MoS_2$ to graphene, which strongly modifies the decay time of both excitonic species. Here, with a similar fitting procedure we find a new value of $\tau_A$ of 3 ns, regardless of $V_G$. This shorter lifetime accounts for the ultrafast charge transfer from the bottom conduction band of $MoS_2$ to graphene. Furthermore, in the HS, increasing $V_{\text{G}}$ =−30 V to $V_{\text{G}}$ =30 V leads to the additional process of hot electron transfer before internal recombination, which is responsible for a similar quenching of the A and B excitons PL intensities by a factor of two. The latter

| **Parameter** | **Type** | **Description** | **Value** | **Unit** |
|---|---|---|---|---|
| $n_{\mathrm{D}}$ | Variable | Doping density in $MoS_2$ | $\sim 2.3 \times 10^{12}$ | $\mathrm{cm}^{-2}$ |
| $E_{in}$ | Fixed | Incident photon energy | 2.33 | eV |
| $S$ | Fixed | Illumination area | $\sim 3.14 \times 10^{-12}$ | $m^2$ |
| $\eta_{\mathrm{A}}$ | Variable | A exciton absorption efficiency | $\sim 0.18$ | - |
| $\eta_{\mathrm{B}}$ | Variable | B exciton absorption efficiency | $\sim 0.18$ | - |
| $\tau_{\mathrm{A}}$ | Variable | A exciton lifetime | $\sim 30$ | ns |
| $\tau_{\mathrm{A}^-}$ | Variable | Trion lifetime | $\sim 4$ | ns |
| $\tau_{\mathrm{B}}$ | Variable | B exciton lifetime | $\sim 0.4$ | ns |
| $\tau_{\mathrm{B}\to\mathrm{A}}$ | Variable | Decay time from B-exciton to A exciton | $\sim 70$ | fs |
| $\tau_{\mathrm{diss}}$ | Variable | Trion dissociation time | $\sim 2$ | fs |
| $k$ | Variable | Trion formation rate | $\sim 3 \times 10^3$ | $\mathrm{cm}^2/\mathrm{s}$ |

TABLE I. Fitting parameters.

manifests in the reduction of $\eta_{\mathrm{A}}$ and $\eta_{\mathrm{B}}$ which in the HS are 0.09, two times smaller than the value 0.18 (Table. I) found in $MoS_2$.

## S3 COMPUTATIONAL METHODS

The calculations carried out in this work were performed in the framework of density functional theory (DFT)[4] using the Vienna ab-initio simulation Package (VASP)[5]. The electron-nuclear interactions in the Kohn-Sham equations[6] are described using the projector augmented wave method[7], while the exchange-correlational potential is treated within the generalized gradient approximation as proposed by Perdew, Burke, and Ernzerhof (PBE)[8]. Van der Waals interactions are incorporated through the Grimme's D3 correction[9]. Spin-orbit coupling is included in the electronic structure calculations. A plane wave energy cutoff of 400 eV is used along with a k-mesh including 6×6×1 points to sample the Brillouin zone. Convergence thresholds of $1 \times 10^{-6}$ eV for the energy and 20 meV/Å for the residual interatomic forces are adopted. A vacuum slab of 18 Å is added in the normal direction of the two-dimensional layers to prevent spurious interactions between the periodic replicas. Crystal structures are visualized using VESTA[10] and the post-processing code VASPKIT[11]

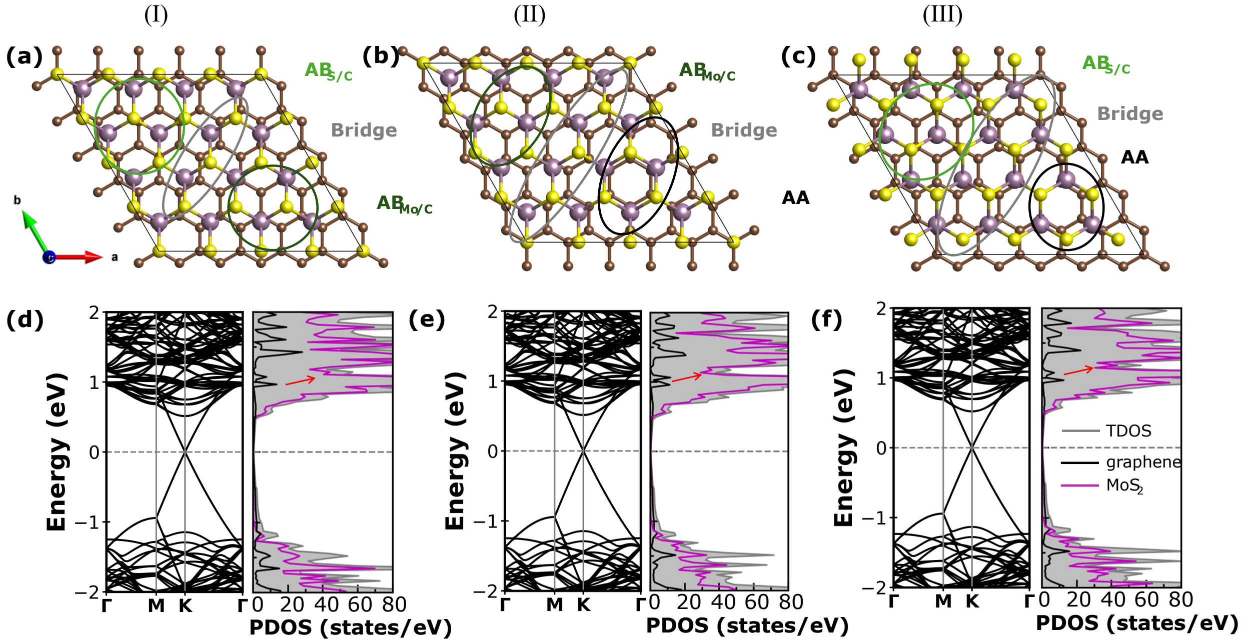


FIG. S2. **Electronic band structures and projected density of states (PDOS) of different superlattices.** (a-c) Atomic arrangements of the three $MoS_2$/graphene superlattices considered in this work with brown, yellow, and violet spheres representing C, S, and Mo atoms, respectively. Local stacking domains, including AA, ABMo/C, ABS/C, and Bridge regions, are circled in black, dark green, light green, and grey colors, respectively. In the AA stacking, both Mo and S atoms are above the C atoms of graphene, whereas in the ABS/C (ABMo/C) configurations, the S (Mo) atoms are atop C atoms with the Mo (S) atoms located at the center of the hexagon. In the bridge regions, no specific ordering is recognizable. (d–f) Electronic band structures and PDOS of the superlattices. Red arrows indicate slight modifications in the $MoS_2$ states due to the presence of different local stackings.

is used to plot the band structures and construct the superlattices.

The superlattices considered in this work consist of a 5×5 graphene supercell and a 4×4 $MoS_2$ supercell, leading to a total of 98 atoms in the simulation box. The adopted setup minimizes the in-plane strain generated by the 22% lattice mismatch between graphene and $MoS_2$ in their unit cells. Given the sensitivity of $MoS_2$ to strain[12,13], the lattice deformation is applied entirely to graphene, which is subject to a 3% compression of its lattice parameter. To explore the effect of a local twisting between the constituting monolayers, we consider three high-symmetry configurations including local domains with so-called AA,

AB, and Bridge stackings. To analyze the role of the mutual arrangement between graphene and $MoS_2$ on the electronic structure of their heterobilayers, we performed ab initio simulations for three superlattices with different stacking configurations, see Fig. S2(a-c) which are representative of local structural domains observed in twisted bilayer systems[14–16]. The considered superlattices consist of a 5×5 graphene supercell and a 4×4 $MoS_2$ supercell. After relaxation, the systems exhibit three distinct types of local domains characterized by AA and AB stackings, as well as by so-called Bridge configurations in which none of the above is recognized. The interlayer distances in these domains range from 3.36 Å to 3.46 Å, in good agreement with earlier ab initio results[16] obtained, however, under slightly different settings and approximations. The electronic properties of the resulting superlattices are nearly identical in the gap region (see Fig. S2(d–f)), which rules the excitonic properties and the optical response of the materials. The Dirac cone of graphene and the band edges of $MoS_2$ can be clearly identified, in agreement with previous findings[16]. Note that the band structures visualized in Fig. S2(d–f) are folded into the Brillouin zone of the superlattices. To reconcile them with the conventional dispersion of monolayer $MoS_2$, an unfolding procedure[17,18] should be applied, which, however, is not relevant for the purpose of this analysis. The main effects of the different local stacking domains are visible in the conduction region around 1.2 eV [red arrows in Fig. S2(d–f)] and affect mostly $MoS_2$ states. Based on these findings, we can safely assume that the presence of local stacking registries induced by twist angles in $MoS_2$/graphene HSs does not significantly affect the charge transfer process.

## REFERENCES


[1] Kim, S.; Nah, J.; Jo, I.; Shahrjerdi, D.; Colombo, L.; Yao, Z.; Tutuc, E.; Banerjee, S. K. Realization of a high mobility dual-gated graphene field-effect transistor with $Al_2O_3$ dielectric. *Appl. Phys. Lett.* **2009**, *94*.

[2] Meric, I.; Dean, C. R.; Petrone, N.; Wang, L.; Hone, J.; Kim, P.; Shepard, K. L. Graphene field-effect transistors based on boron–nitride dielectrics. *Proc. IEEE* **2013**, *101*, 1609–1619.

[3] Petrone, N.; Chari, T.; Meric, I.; Wang, L.; Shepard, K. L.; Hone, J. Flexible graphene field-effect transistors encapsulated in hexagonal boron nitride. *ACS nano* **2015**, *9*, 8953–8959.

[4] Hohenberg, P.; Kohn, W. Inhomogeneous electron gas. *Phys. Rev.* **1964**, *136*, B864.

[5] Kresse, G.; Furthmüller, J. Efficient iterative schemes for ab initio total-energy calculations using a plane-wave basis set. *Phys. Rev. B* **1996**, *54*, 11169.

[6] Kohn, W.; Sham, L. J. Self-consistent equations including exchange and correlation effects. *Phys. Rev.* **1965**, *140*, A1133.

[7] Blöchl, P. E. Projector augmented-wave method. *Phys. Rev. B* **1994**, *50*, 17953.

[8] Perdew, J. P.; Burke, K.; Ernzerhof, M. Generalized gradient approximation made simple. *Phys. Rev. Lett.* **1996**, *77*, 3865.

[9] Grimme, S.; Antony, J.; Ehrlich, S.; Krieg, H. A consistent and accurate ab initio parametrization of density functional dispersion correction (DFT-D) for the 94 elements H-Pu. *J. Chem. Phys.* **2010**, *132*.

[10] Momma, K.; Izumi, F. VESTA 3 for three-dimensional visualization of crystal, volumetric and morphology data. *J. Appl. Crystallogr.* **2011**, *44*, 1272–1276.

[11] Wang, V.; Xu, N.; Liu, J.-C.; Tang, G.; Geng, W.-T. VASPKIT: A user-friendly interface facilitating high-throughput computing and analysis using VASP code. *Comput. Phys. Commun.* **2021**, *267*, 108033.

[12] Blundo, E.; Felici, M.; Yildirim, T.; Pettinari, G.; Tedeschi, D.; Miriametro, A.; Liu, B.; Ma, W.; Lu, Y.; Polimeni, A. Evidence of the direct-to-indirect band gap transition in strained two-dimensional $WS_2$, $MoS_2$, and $WSe_2$. *Phys. Rev. Research* **2020**, *2*, 012024.

[13] Ramzan, M. S.; Cocchi, C. Strained monolayer MoTe2 as a Photon Absorber in the Telecom

range. *Nanomaterials* **2023**, *13*, 2740.

[14] Liu, X.; Balla, I.; Bergeron, H.; Campbell, G. P.; Bedzyk, M. J.; Hersam, M. C. Rotationally commensurate growth of $MoS_2$ on epitaxial graphene. *ACS nano* **2016**, *10*, 1067–1075.

[15] Lu, C.-I.; Butler, C. J.; Huang, J.-K.; Chu, Y.-H.; Yang, H.-H.; Wei, C.-M.; Li, L.-J.; Lin, M.-T. Moiré-related in-gap states in a twisted $MoS_2$/graphite heterojunction. *npj 2D Mater. Appl.* **2017**, *1*, 24.

[16] Ramzan, M. S.; Goodwin, Z. A.; Mostofi, A. A.; Kuc, A.; Lischner, J. Effect of Coulomb impurities on the electronic structure of magic angle twisted bilayer graphene. *npj 2D Mater. Appl.* **2023**, *7*, 49.

[17] Boykin, T. B.; Klimeck, G. Practical application of zone-folding concepts in tight-binding calculations. *Phys. Rev. B:Condens. Matter Mater. Phys.* **2005**, *71*, 115215.

[18] Krumland, J.; Cocchi, C. Conditions for electronic hybridization between transition-metal dichalcogenide monolayers and physisorbed carbon-conjugated molecules. *Electron. Struct.* **2021**, *3*, 044003.
